# Vitiligo révélant une maladie de Vogt-Koyanagi-Harada

**DOI:** 10.11604/pamj.2017.27.220.11656

**Published:** 2017-07-24

**Authors:** Mohamed El Amraoui, Youssef Zemmez, Ahmed Bouhamidi, Rachid Frikh, Naoufal Hjira, Mohammed Boui

**Affiliations:** 1 Service de Dermatologie-Vénéréologie, Hôpital Militaire d’Instruction Mohammed V, Rabat, Maroc

**Keywords:** Vitiligo, Vogt-Koyanagi-Harada, auto-immunité, mélanocyte, lymphocyte T, Vitiligo, Vogt-Koyanagi- Harada, autoimmunity, melanocyte, T lymphocyte

## Abstract

Le vitiligo est une dermatose chronique auto-immune, souvent associé ou fait découvrir d'autres pathologies auto-immunes. Son association à une atteinte ophtalmologique à type de pan uvéite et /ou une atteinte neurologique à type de méningite et/ou de l'oreille interne à type de surdité détermine la maladie ou le syndrome de Vogt-Koyanagi-Harada (VKH). Nous rapportons le cas d'une jeune femme qui consultait pour des uvéites récidivantes depuis un an, Et ce n'était qu'avec l'apparition des lésions de vitiligo que le diagnostic de maladie de VKH a été évoqué et confirmé.

## Abstract (en)

Vitiligo is a chronic auto-immune skin disease, often associated or discovers other autoimmune pathologies. His association with Ophthalmological type pan uveitis and/or neurological type of meningitis and/or inner ear type of hearing loss determines the disease or Vogt -Koyanagi-Harada syndrome (VKH). We related the case of a young woman who consulted for recurrent uveitis for a year, and it was only with the onset of vitiligo lesions that VKH disease diagnosis was discussed and confirmed.

## Introduction

Le vitiligo est une dermatose chronique auto-immune, souvent associé ou fait découvrir d'autres pathologies auto-immunes. Son association à une atteinte ophtalmologique à type de pan uvéite et /ou une atteinte neurologique à type de méningite et/ou de l'oreille interne à type de surdité détermine la maladie ou le syndrome de Vogt-Koyanagi-Harada (VKH). Nous rapportons le cas d'une jeune femme qui consultait pour des uvéites récidivantes depuis un an, Et ce n'était qu'avec l'apparition des lésions de vitiligo que le diagnostic de maladie de VKH a été évoqué et confirmé.

## Patient et observation

**Figure 1:**
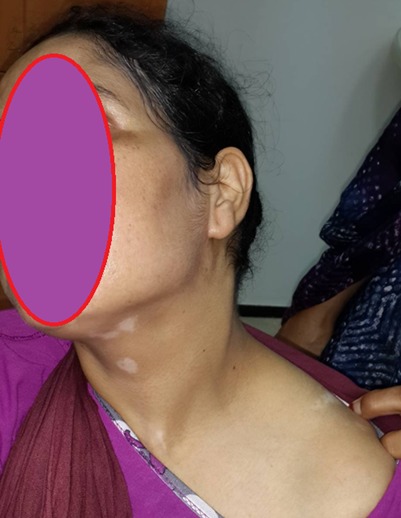
Lésions de vitiligo avec poliose du cuir chevelu

**Figure 2:**
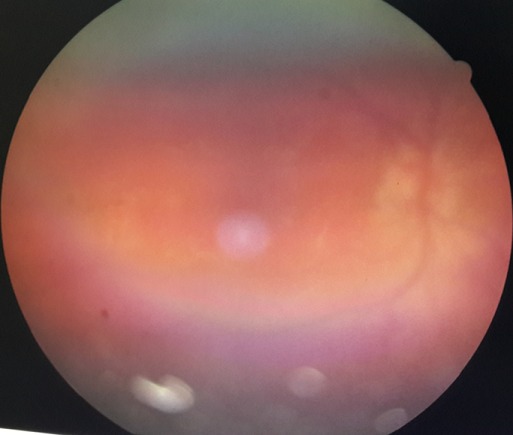
Rétinographie: péri-vascularite témoignant d’une phlébite postérieure dans le cadre d’une uvéite postérieure

**Figure 3:**
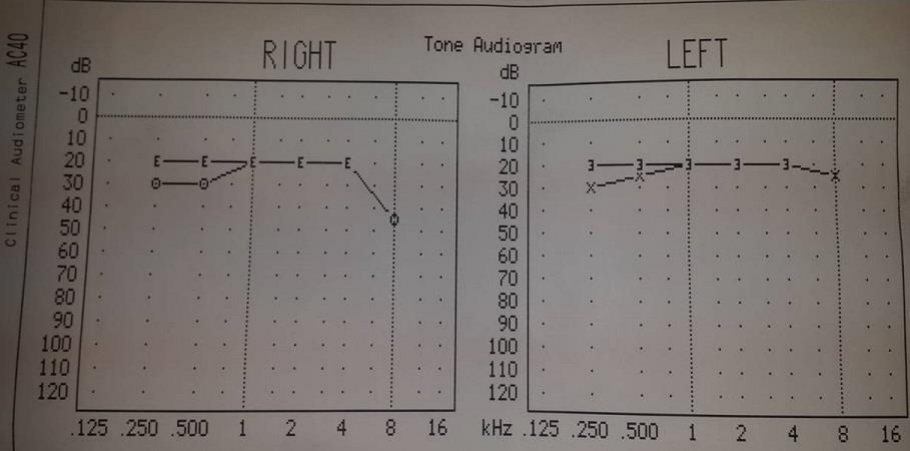
Audiogramme de la patiente objectivant une discrète surdité

Patiente âgée de 31 ans, suivie en Ophtalmologie pour une pan uvéite évoluant par poussées et remissions depuis un an, a consulté pour des lésions de vitiligo siégeant au niveau du cou et évoluant depuis trois mois, avec une poliose du cuir chevelu sans alopécie (Figure 1). L'examen ophtalmologique montrait une baisse profonde de l'acuité visuelle des deux yeux, le tonus oculaire était normal, l'examen des annexes montrait des cils de coloration normale sans vitiligo au niveau des paupières, l'examen du segment antérieur montrait des précipités retro dyscinétiques en graisse de mouton sans Tyndall de la chambre antérieure avec des synéchies irido-cristalliniennes et une cataracte bilatérale, le fond d'œil était très difficile à cause d'un myosis très serré, les rétinographies montraient un décollement rétinien de l'œil droit et des lésions rétiniennes de l'œil gauche, une péri-vascularite témoignant d'une phlébite postérieure dans le cadre d'une uvéite postérieure (Figure 2). L'examen ORL objectivait une discrète surdité (Figure 3). L'examen neurologique était strictement normal et les examens clinique et para clinique à la recherche d'autres maladie auto-immunes associées étaient négatifs. La patiente a été mise sous corticothérapie orale et topique avec une évolution favorable.

## Discussion

La maladie de Harada ou syndrome de Vogt-Koaynagi-Harada, décrite par Vogt en 1906, Harada en 1926 et Koyanagi en 1929, est une pan uvéite granulomateuse bilatérale, chronique et diffuse, caractérisée par un décollement rétinien séreux et fréquemment associée à des atteintes neurologiques, auditives et dermatologiques [1]. Son incidence est estimée à 1/400 000 habitants avec une répartition géographique variable, au Japon elle représente près de 8% des uvéites. La maladie affecte essentiellement les jeunes femmes mélanodermes. Les enfants peuvent être affectés, mais l'âge moyen du début de la maladie est plutôt de 30 ans (de 10 à 52 ans) [2,3]. La pathogenèse de l'affection a été reliée à un désordre immunologique dirigé contre les mélanocytes entraînant une cytotoxicité et une apoptose médiées par les lymphocytes T. Les mélanocytes étant des cellules originaires de la crête neurale et forment la peau, les méninges, la rétine, l'uvée, la cochlée et le labyrinthe et donc la maladie peut toucher ces différents organes. Cette maladie auto-immune qui se développerait sur un terrain génétique prédisposant est fréquemment associée avec l'haplotype DRB1*0405 [4, 5]. Cliniquement, la maladie évolue selon quatre phases: **la phase prodromique**: est caractérisée par des symptômes non spécifiques tels qu'une fièvre, des maux de tête, des nausées et des vertiges, puis par des symptômes neurologiques tels qu'une faiblesse musculaire, une hémiparésie, une hémiplégie, une dysarthrie et une douleur orbitale. Durant **la phase ophtalmologique**: qui survient quelques jours après la phase prodromique, les patients souffrent de troubles visuels, de douleur oculaire, de photophobie ou de scotome central (bilatéral dans 80 % des cas). Un décollement rétinien bilatéral et séreux survient fréquemment. Une perte de l'audition et des vertiges peuvent aussi être présents. **La phase de convalescence** qui survient dans les 3 mois après le début de la maladie, est caractérisée par l'apparition de signes cutanés tels qu'une poliose affectant les cils et les sourcils (parfois les cheveux), une chute de cheveux et un vitiligo. Une uvéite récurrente et des complications ophtalmologiques apparaissent dans la dernière phase de la maladie, **la phase récurrente chronique** . On peut voir apparaître des néo-vaisseaux choroïdiens, un glaucome ou une cataracte secondaire [6].

Des critères diagnostiques ont été établis et permettent de poser le diagnostic de la maladie: **les critères de Sugiura (1978)** comportaient trois symptômes majeurs pour porter le diagnostic de VKH: uvéite antérieure bilatérale, décollements séreux rétiniens et pléiocytose. Les autres symptômes de la maladie (hypoacousie, vertiges, alopécie, poliose, vitiligo et dépigmentation du fond d'oeil) étaient considérés mineurs. **Les critères de « l'American Uveitis Society » (1980)** ont fait l'objet d'une révision en 2001 et permettent de classer les formes de la maladie selon leur caractère « complet », incomplet » ou « probable ». D'après la littérature, il existe une concordance globale satisfaisante entre les classifications pour le diagnostic de VKH, les critères de Sugiura sont performants pour le diagnostic des patients à la phase aiguë ou lors des récurrences, Inversement, les critères de « l'American Uveitis Society » permettent de porter plus aisément le diagnostic des formes chroniques [7]. Le diagnostic de la maladie de Vogt-Koyanagi-Harada est essentiellement clinique, les examens complémentaires contribuent au diagnostic de la maladie dans les formes incomplètes et atypiques et ils sont de deux types, ceux explorant l'atteinte oculaire (angiographie à la fluorescéine, tomographie en cohérence optique (OCT), échographie oculaire en mode B…), et ceux explorant les autres manifestations extra oculaires (ponction lombaire, IRM cérébrale, audiogramme…) [8]. Les diagnostics différentiels peuvent être discutés en fonction de la présentation clinique de la maladie, ainsi, Il faut différencier cette maladie d'une choriorétinopathie séreuse centrale, des métastases choroïdiennes, d'une ophtalmie sympathique, des autres causes des uvéites, du syndrome d'Alezzandrini ( hypoacousie précédant la surdité, rétinite pigmentaire, vitiligo et poliose d'un seul coté) et du syndrome Retinitis pigmentosa associated with hearing loss, thyroid disease, vitiligo, and alopecia areata. Le traitement corticoïde à fortes doses est en général efficace et doit être prolongé pour éviter les rechutes, les immunosuppresseurs et les biothérapies peuvent également être utilisées. Si la prise en charge est précoce et le traitement est agressif, le pronostic est en général favorable, mais une altération sévère de l'audition et de la vue peut survenir [9].

## Conclusion

La maladie de Vogt-Koyanagi-Harada est une maladie auto-immune dirigée contre les mélanocytes et peut toucher alors l'uvée, les méninges, l'oreille interne et la peau. Sa prise en charge doit faire intervenir une équipe pluridisciplinaire associant ophtalmologiste, neurologue, ORL et dermatologue à fin de prévenir certaines complications compromettant le pronostic fonctionnel plus que vital.

## Conflits d’intérêts

Les auteurs ne déclarent aucun conflit d'intérêts.
